# P-1437. The Effect of Pre-Vaccination Analgesics on Influenza Vaccine Immunogenicity

**DOI:** 10.1093/ofid/ofaf695.1624

**Published:** 2026-01-11

**Authors:** Carlie N Skellington, Kat Schmidt, Christina Schofield, Anuradha Ganesan, Wesley Campbell, Tahaniyat Lalani, Katrin Mende, Ana E Markelz, Adam Saperstein, Drake Tilley, Alan Williams, Derek T Larson, Laurie Housel, Bruce McClenathan, Srihari Seshadri, Simon Pollett, Timothy Burgess, Stephanie A Richard, Rhonda E Colombo

**Affiliations:** Madigan Army Medical Center, Tacoma, WA, USA, TACOMA, WA; Infectious Disease Clinical Research Program, USUHS, Arlington, Virginia; Madigan Army Medical Center, Tacoma, Washington; Infectious Disease Clinical Research Program, USUHS; Henry M. Jackson Foundation for the Advancement of Military Medicine Inc, Bethesda, Maryland; Walter Reed National Military Medical Center, Bethesda, Maryland; Naval Medical Center Portsmouth, Portsmouth, Virginia; 1Infectious Disease Clinical Research Program, Department of Preventive Medicine and Biostatistics, Uniformed Services University of the Health Sciences and Brooke Army Medical Center, JBSA Fort Sam Houston, TX, San Antonio, TX; Brooke Army Medical Center, San Antonio, Texas; USUHS, Bethesda, Maryland; Naval Health Clinic Annapolis, Annapolis, MD, USA, Annapolis, Maryland; Uniformed Services University of the Health Sciences, Bethesda, Maryland; Naval Medical Center San Diego and Uniformed Services University, SAN DIEGO, CA; Womack Army Medical Center, Fort Liberty, North Carolina; Womack Army Medical Center, Fort Liberty, North Carolina; Defense Health Agency, Springfield, Virginia; Infectious Disease Clinical Research Program, Department of Preventive Medicine and Biostatistics, Uniformed Services University of the Health Sciences, Bethesda, MD, USA, Bethesda, Maryland; Infectious Disease Clinical Research Program, Department of Preventive Medicine and Biostatistics, Uniformed Services University of the Health Sciences, Bethesda, MD, USA, Bethesda, Maryland; Infectious Disease Clinical Research Program, Department of Preventive Medicine and Biostatistics, Uniformed Services University of the Health Sciences, Bethesda, MD, USA, Bethesda, Maryland; HJF/USU IDCRP, Tacoma, Washington

## Abstract

**Background:**

Analgesics are sometimes taken prior to vaccination to prevent adverse reactions. Although several studies have examined the immunogenicity impact of post-vaccination analgesics, few have assessed the effect of analgesics taken prior to vaccination. This study compares antibody responses to hemagglutination (HA) among adults with and without analgesic use prior to influenza vaccine receipt. It further analyzes differences in immunogenicity according to analgesic type.

**Figure:**
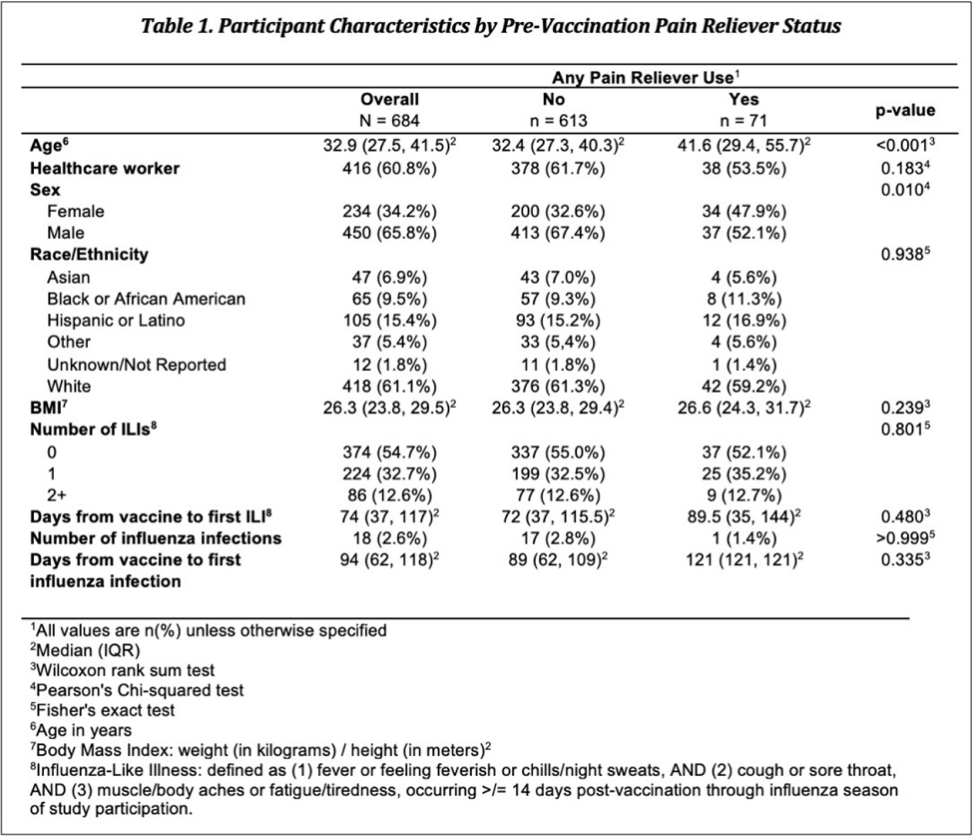

**Figure:**
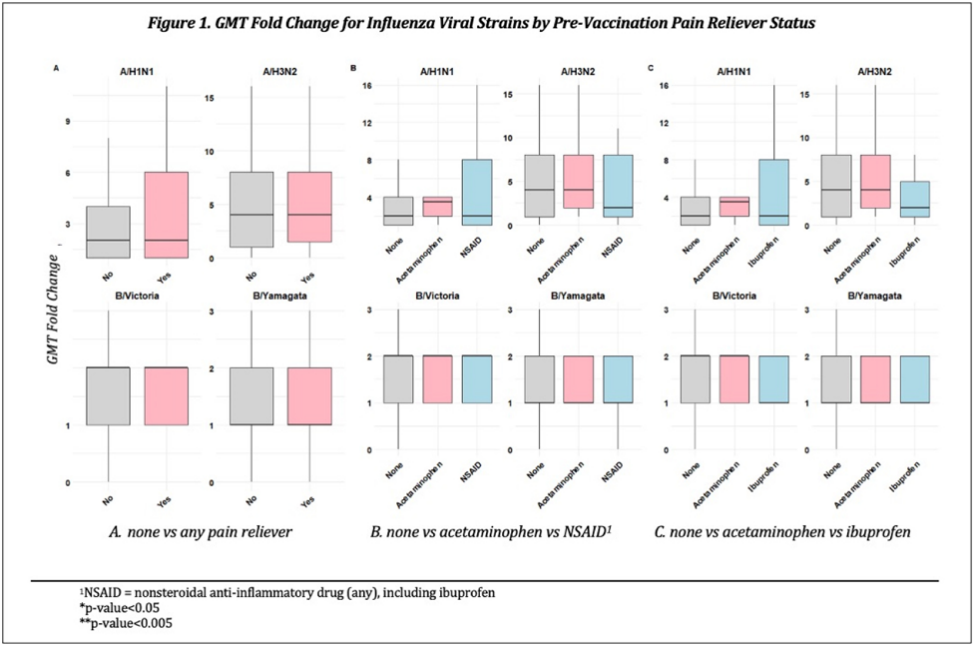

**Methods:**

Pragmatic Assessment of Influenza Vaccine Effectiveness in the DoD (PAIVED) was an open-label, randomized clinical trial comparing the effectiveness of FDA-licensed influenza vaccines on adult military healthcare beneficiaries. A subset of participants provided venous blood samples collected pre-vaccination and 30 days post-vaccination. Participants included in this analysis provided venous blood samples and reported on analgesic use within 24 hours prior to vaccine receipt.

**Figure:**
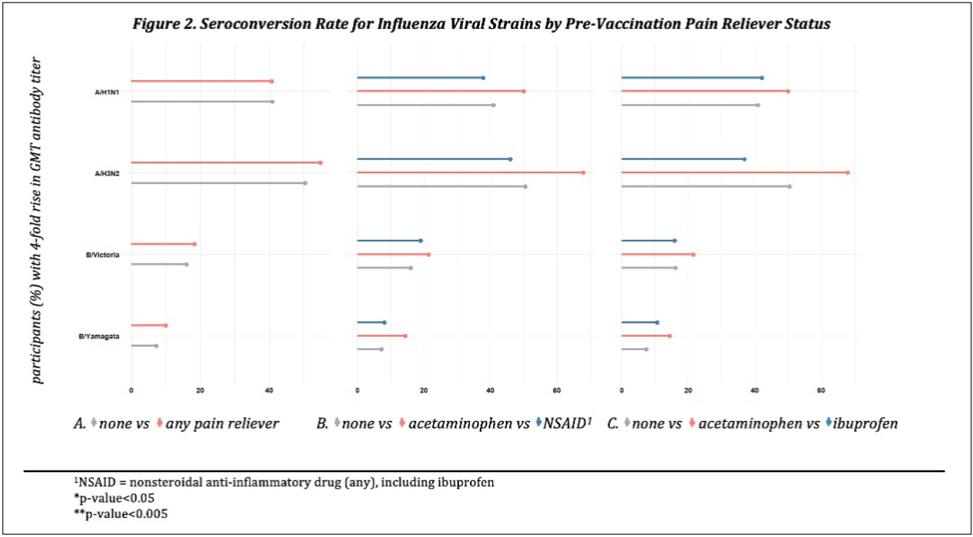

**Results:**

Of 1,173 PAIVED participants with pre- and post-vaccination anti-HA antibody titers, 684 (58%) answered the analgesic question. Among those, 71 (10%) reported taking an analgesic within 24 hours prior to vaccine receipt. The most prevalent analgesics were nonsteroidal anti-inflammatory drugs (NSAIDs) including ibuprofen (total NSAIDs, n=37; ibuprofen, n=19) and acetaminophen (n=28). Those who took a pre-vaccination analgesic were significantly older and more often female (Table 1). There were no significant differences in the average change in geometric mean titer (GMT) among subjects with a pre-vaccination analgesic compared to those without, including when stratified by analgesic type (Figure 1). While a higher percentage of people who took acetaminophen seroconverted (4-fold rise in antibody titer) compared to those who took ibuprofen or other NSAIDs, this finding was not statistically significant (Figure 2).

**Conclusion:**

Overall, pre-vaccination analgesic use in PAIVED did not have a significant impact on influenza vaccine immunogenicity, although there was a suggestion that acetaminophen may have a more favorable effect on anti-HA seroconversion than NSAIDs for some vaccine strains. Assessment of the interplay among factors potentially influencing these findings may provide useful insight.

**Disclosures:**

All Authors: No reported disclosures

